# Supplementary material for: The causal relationship between the human gut microbiota and pyogenic arthritis: a Mendelian randomization study
Source: Front Cell Infect Microbiol. 2024 Nov 26;14:1452480. doi: 10.3389/fcimb.2024.1452480 (PMC11629706; doi:10.3389/fcimb.2024.1452480)
Supplement: Supplementary file 1 [file DataSheet1.zip › mendelian test/ebi-a-GCST90027641.csv_leave_one_out.pdf]

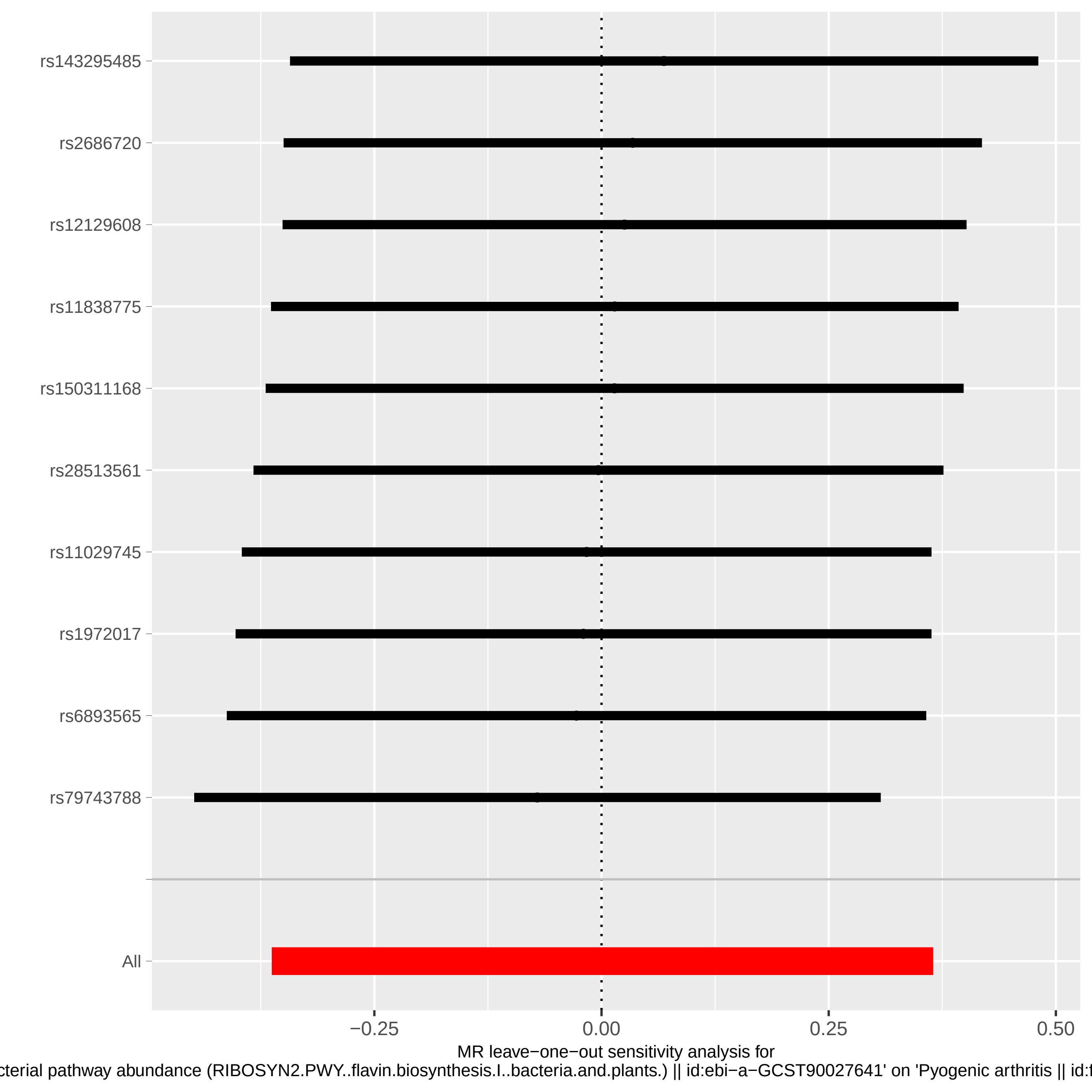
rs143295485
rs2686720
rs12129608
rs11838775
rs150311168
rs28513561
rs11029745
rs1972017
rs6893565
rs79743788
All
−0.25
0.00
0.25
0.50
MR leave−one−out sensitivity analysis for
cterial pathway abundance (RIBOSYN2.PWY..flavin.biosynthesis.I..bacteria.and.plants.) || id:ebi−a−GCST90027641' on 'Pyogenic arthritis || id:f
